# CY-TB skin test: Detect and defeat

**DOI:** 10.6026/973206300212218

**Published:** 2025-07-31

**Authors:** Aditi Bharti, Tej Pratap Singh

**Affiliations:** 1 Department of Community and Family Medicine, AIIMS Bhopal, Madhya Pradesh, India; 2 Department of Community Medicine, Sukh Sagar Medical College and Hospital, Jabalpur, Madhya Pradesh, India

**Keywords:** Latent Tuberculosis Infection (LTBI), Cy-Tb, Tuberculin Skin Test (TST), Interferon γ Release Assay (IGRA), Tuberculosis Preventive Treatment (TPT)

## Abstract

Latent Tuberculosis Infection (LTBI) is a sustained immune response to Mycobacterium tuberculosis without clinical evidence of active disease, representing a substantial reservoir, contributes to the global burden of tuberculosis (TB). To address these gaps, Cy-Tb test (formerly C-Tb), developed by the Serum Institute of India, Pune, has been introduced under NTEP policy, combines the operational simplicity of TST with the specificity of IGRAs, expanded the coverage of high-risk groups and household contacts of pulmonary TB patients. Cy-Tb represents a next-generation LTBI diagnostic solution that supports early detection, operational alignment with point-of-care testing, cost-effectiveness, integration potential into national programs and contributes significantly towards the goal of TB elimination in India by 2025 and globally by 2035.

## Background:

Latent tuberculosis bacterial infections (LTBI) are a significant challenge to pursue as a state of persistent immune response to Mycobacterium tuberculosis antigens with no evidence of clinical manifestations [1]. LTBI treatment has an imperative role in mitigating tuberculosis and is a crucial pillar in the achievement of NSP, SDG and WHO's End TB Strategy. This lowers the risk of developing LTBI into active tuberculosis, which is a kind of risk-benefit trade-off. Therefore, developing the advancements with high accuracy and specificity is a key challenge to deal with [2, 3, 4-5]. The development of the tuberculin skin test (TST) has been the standard method to dignify with the test for M. tuberculosis, initiated in 1908 to curtail the risk of LTBI from active tuberculosis [6-7]. Nevertheless, important limitations of the TST are said to escalate the number of false-positive reactions that occur in individuals infected with nontuberculous mycobacteria and with previous BCG vaccination. Therefore, the implementation of the interferon-γ release assays (IGRAs), which is based on the highly immunogenic antigens, *i.e.*, specific to M. tuberculosis, formulated under the NTEP policy to curtail the issues of the interaction with the BCG vaccine and infection with nontuberculous mycobacteria, has previously been noticed with the TST. Moreover, its complications in terms of laboratory infrastructure and skilled staff lower down the execution of IGRAs at an expanded coverage in a way that could be more meaningful [8-9]. Despite the overwhelming formulation of IGRA with its more comprehensibility, it failed to express the variable coverage concerning household contacts and high-risk detection. Hence, in line with the commitment to end TB in India by 2025, NTEP formulated a novel policy in 2021 to amplify the coverage of pulmonary TB household contact patients and high-risk populations through Tuberculosis Preventive Treatment (TPT), with the introduction of Cy-Tb, designed holistically to achieve the operational advantages [10]. Therefore, it is of interest to describe how this research will provide the insight about the importance of CY-tb to strengthen the programmatic uptake through collaborative approach in detecting, diagnosing, and defeating TB.

## Cy-TB: Diagnose, Detect & Defeat:

Worldwide, among 25% of the estimated population, and nationally, according to the National Prevalence Survey 2019-21, around 31.4% are infected with latent TB [10], where 5-10% of the LTBI develops into active tuberculosis, which eventually hinders breaking the chain of transmission and facilitates community spread. Thus, detecting latent TB to defeat active TB is the cornerstone of the TB elimination strategy. Cy-TB providing the point-of-care test to facilitate with accuracy at any level, *i.e.*, at the community, hospital, clinic, camp, or national level, through which LTBI detection would not be a challenge anymore [11, 12-13]. However, every new field test has some sort of advantages and disadvantages to overcome, along with new challenges to face. Therefore, hereby presenting the SWOT analysis, which have developed with a focus on changing the scenarios of minimizing active tuberculosis in terms of upscaling the TPT coverage with timely diagnosis and adequate treatment regimen, with new opportunities and curtailing the possible challenges shown in Figure 1 (see PDF).

## Discussion:

In line with the commitment to end TB as a public health problem by 2025, ahead of the global End TB Strategy timeline of 2035, a policy was formulated in 2021 to expand the coverage of TPT under NTEP for all household contacts and bridge the population to overcome LTBI. With this objective in mind, the new advancement has been enhanced as the Cy-TB skin test to detect all possible contacts of the TB index case with minimal resources. This research facilitates the comprehensibility concerning Cy-TB as a detecting tool to defeat LTBI over TST and IGRA. The combination of operational simplicity and functional ability with possible resources initiates the formulation and feasibility of this new development. From a peripheral standpoint, Cy-Tb offers multiple advantages over the prior test [11], which makes this especially suitable for decentralized use in the community or peripheral health centres. On another note, cost-effectiveness is indeed a vital advantage. A recent economic modelling study in India evaluated the use of Cy-Tb for LTBI screening and found it to be a cost-effective strategy for preventing future TB cases. By avoiding the high laboratory costs associated with IGRAs and leveraging existing training from decades of TST use, Cy-Tb can deliver accurate results at a fraction of the cost of current IGRA tests [14]. By expanding LTBI testing coverage with a more practical tool, the program aims to offer Tuberculosis Preventive Treatment (TPT) too many more people who need it. Likewise, household contacts of pulmonary TB patients who are at high risk of harbouring latent infection can be tested with Cy-Tb, and those who test positive can be started on preventive therapy promptly. This targeted approach will help intercept potential TB cases before they emerge, thereby reducing the upcoming burden of disease [4, 10]. In essence, deploying Cy-Tb widely empowers a proactive strategy: detect latent TB now to defeat active TB later, with an approach to accelerate the progress towards the elimination. Overall, this novel step-forward skin test represents a significant advancement in India's fight against tuberculosis. It addresses previous diagnostic gaps by providing a more accurate yet feasible method to identify latent infections. With strong political will and programmatic support, Cy-Tb has the potential to substantially escalate the uptake of TB preventive therapy, thereby shrinking the reservoir of infection that has long sustained TB transmission. The breakthrough commitment shown by the Government of India, including validation of Cy-Tb through the Indian Council of Medical Research (ICMR) and rapid incorporation into national policy. If effectively implemented and facilitated, Cy-Tb could be a game-changer in accelerating progress toward a TB-free India, serving as a model for other high-burden countries. By combining improved LTBI detection with scaled-up preventive treatment, India is moving decisively to detect, diagnose, and defeat TB at all levels [10, 14].

## Conclusion:

Achieving the objective to eliminate tuberculosis by 2025 and the WHO End TB Strategy by 2035, changing the current situation is mandatory, where 5-10% of the LTBI develop active tuberculosis, which is a remarkable hindrance in the elimination of TB. Hence, launching and executing Cy-TB is the crucial step taken by the Government of India through the validation of the ICMR as a cost-effective and life-saving strategy, which exemplifies our unwavering dedication to TB eradication globally.

## Way forward:

Implementing the Cy-TB proves to be a cornerstone in the control and elimination of tuberculosis. Nevertheless, the execution of this test with intensive usage at all levels is still a challenge, which has to be carried out by integrating Cy-TB into the Ni-kshay, healthcare professionals, diagnostic centres, and laboratories to participate actively in the submission of Cy-TB results, ensuring the timely detection of LTBI, commencing the required treatment, and also escalating the coverage of TPT. This exercise will eventually decrease the significant burden of TB.
